# Effect of Two Lipoprotein (a)-Associated Genetic Variants on Plasminogen Levels and Fibrinolysis

**DOI:** 10.1534/g3.116.034702

**Published:** 2016-09-06

**Authors:** Hong Wang, Chan E. Hong, Joshua P. Lewis, Yanbei Zhu, Xing Wang, Xin Chu, Joshua Backman, Ziying Hu, Peixin Yang, Christopher D. Still, Glenn S. Gerhard, Mao Fu

**Affiliations:** * Division of Endocrinology, Diabetes, and Nutrition, University of Maryland School of Medicine, Baltimore, Maryland 21201; † Department of Orthopedic Surgery, Second Affiliated Hospital of Chongqing Medical University, 400010, China; ‡ Geisinger Obesity Institute, Geisinger Clinic, Danville, Pennsylvania 17822; § Department of Obstetrics, Gynecology, & Reproductive Sciences, University of Maryland School of Medicine, Baltimore, Maryland 21201; ** Penn State Institute for Personalized Medicine, Penn State College of Medicine, Hershey, Pennsylvania 17033

**Keywords:** lipoprotein (a), plasminogen, fibrinolysis, genetics, thrombogenicity

## Abstract

Two genetic variants (rs3798220 and rs10455872) in the apolipoprotein (a) gene (*LPA*) have been implicated in cardiovascular disease (CVD), presumably through their association with lipoprotein (a) [Lp(a)] levels. While Lp(a) is recognized as a lipoprotein with atherogenic and thrombogenic characteristics, it is unclear whether or not the two Lp(a)-associated genetic variants are also associated with markers of thrombosis (*i.e.*, plasminogen levels and fibrinolysis). In the present study, we genotyped the two genetic variants in 2919 subjects of the Old Order Amish (OOA) and recruited 146 subjects according to the carrier and noncarrier status for rs3798220 and rs10455872, and also matched for gender and age. We measured plasma Lp(a) and plasminogen levels in these subjects, and found that the concentrations of plasma Lp(a) were 2.62- and 1.73-fold higher in minor allele carriers of rs3798220 and rs10455872, respectively, compared with noncarriers (*P* = 2.04 × 10^−17^ and *P* = 1.64 × 10^−6^, respectively). By contrast, there was no difference in plasminogen concentrations between carriers and noncarriers of rs3798220 and rs10455872. Furthermore, we observed no association between carrier status of rs3798220 or rs10455872 with clot lysis time. Finally, plasminogen mRNA expression in liver samples derived from 76 Caucasian subjects was not significantly different between carriers and noncarriers of these two genetic variants. Our results provide further insight into the mechanism of action behind two genetic variants previously implicated in CVD risk and show that these polymorphisms are not major modulating factors for plasma plasminogen levels and fibrinolysis.

Cardiovascular disease is one of the leading causes of morbidity and mortality in the world. While progression of CVD is multifactorial, substantial evidence has shown that lipoprotein (a) [Lp(a)] is a significant and independent risk factor in the development of cardiovascular diseases (Berglund and Ramakrishnan 2004; Danesh *et al.* 2000; Kamstrup *et al.* 2009). Plasma Lp(a) concentration varies up to 1000-fold among individuals, is highly heritable, and is influenced minimally by environmental factors (Hobbs and White 1999; McCormick 2004). Levels of plasma Lp(a) are regulated, in part, by the *LPA* gene located on chromosome 6q26–27, which encodes for apo(a) (Clarke *et al.* 2009). The number of kringle 4 type 2 (KIV-2) repeats can vary from 12 to 51 resulting in 34 apo(a) isoforms with different sizes, and the size of apo(a) is inversely related to the plasma Lp(a) concentration (Gavish *et al.* 1989; Lackner *et al.* 1993). In addition, single nucleotide polymorphisms (SNPs) in the *LPA* gene are associated with Lp(a) levels. Among them, a nonsynonymous SNP that results in an isoleucine to methionine substitution at position 1891 (rs3798220) and an intronic variant (rs10455872) have been confirmed to be strongly associated with increased levels of plasma Lp(a) and the risk of cardiovascular disease (Clarke *et al.* 2009; Li *et al.* 2011; Thanassoulis *et al.* 2013). Indeed, a recently published study by our group has provided additional evidence that these variants are significantly associated with Lp(a)-cholesterol levels independently of each other and KIV-2 repeat number (Lu *et al.* 2015). The elucidation of a potential mechanism of action behind these Lp(a)-associated variants for CVD could lead to novel targets for treatment and/or prevention of CVD.

Lp(a) is recognized as a lipoprotein with atherogenic and thrombogenic characteristics (Caplice *et al.* 2001; Grainger *et al.* 1993; Hajjar *et al.* 1989; Marcovina and Koschinsky 2003). Structurally, Lp(a) is a lipoprotein particle consisting of apolipoprotein (a) [apo(a)] covalently bound to the apolipoprotein (B) (apoB) of an LDL-like particle (Koschinsky *et al.* 1993). Previous studies have shown that apo(a) shares structural homology with plasminogen, including a kringle 4 domain, a kringle 5 domain, and an inactive protease domain (Hancock *et al.* 2003; McLean *et al.* 1987). Plasminogen, a critical protein in fibrinolysis, binds to lysine residues present on fibrin via its kringle domains and gets activated to plasmin by tissue plasminogen activator (tPA) or urokinase (Plow and Hoover-Plow 2004). Due to the structural similarity to plasminogen and the lack of proteolytic activity, it has been suggested that Lp(a) competes with plasminogen for fibrin binding, ultimately resulting in impaired fibrinolysis (Atsumi *et al.* 1998; Hervio *et al.* 1995). Furthermore, plasma plasminogen concentrations vary by about twofold among healthy individuals. The heritability of plasminogen is estimated to range from 0.48 to 0.68 (*Ma et al.* 2014). The plasminogen gene (*PLG*) and *LPA* gene are located on chromosome 6q26 within ∼40 kb of each other (Crawford *et al.* 2008). A recent genome-wide association study (GWAS) has identified nine SNPs within the *LPA* and *PLG* gene region on chr6q26 to be significantly associated with plasminogen levels (Ma *et al.* 2014). Moreover, Lp(a) and plasminogen are primarily produced in the liver and transported into the circulation (Koschinsky *et al.* 1993; Rainwater and Lanford 1989; Saito *et al.* 1980). We speculate that *LPA* and *PLG* mRNA expressions are likely to be coregulated in the liver. The genetic variants within the *LPA* and *PLG* gene region on chr6q26 may regulate Lp(a) and plasminogen levels to contribute to thrombogenicity. This promotes persistence of the clot and the thrombotic process that may contribute to thrombo-atherogenic diseases.

*LPA* SNPs rs3798220 and rs10455872 are consistently associated with Lp(a) levels and result in increased risk for cardiovascular diseases (Arsenault *et al.* 2014; Clarke *et al.* 2009; Kutikhin *et al.* 2014; Thanassoulis *et al.* 2013). However, the mechanism of action behind these two genetic variants previously implicated in CVD risk is not known. In an attempt to better understand the relationship between Lp(a)-associated genetic variants and thrombogenesis we genotyped the two variants in 2919 Amish subjects and recruited 146 age- and sex-matched subjects by rs3798220 and rs10455872 genotype. We measured the levels of plasma Lp(a) and plasminogen and compared the levels of Lp(a) and plasminogen based on rs3798220 and rs10455872 genotypes. In addition, we also assessed genotype-specific differences in fibrinolysis using a euglobulin clot lysis assay (ECLA). Finally, genotype-specific differences in *PLG* mRNA expression were evaluated in 76 liver samples derived from Caucasian subjects.

## Materials and Methods

### Subjects and genotyping

**Figure 1:**
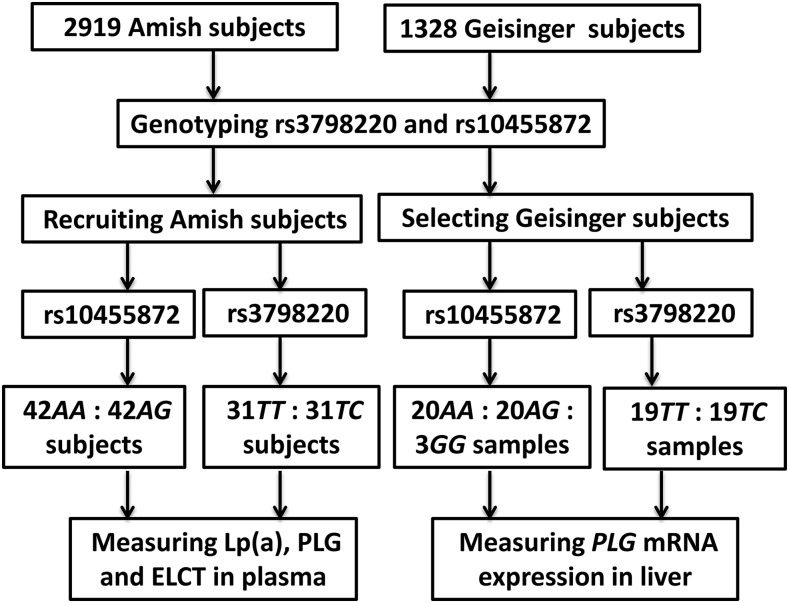
A flowchart of study design.

The subjects were from the Old Order Amish community (OOA) of Lancaster, PA and were drawn from participants of our previous Lp(a) study (Lu *et al.* 2015), the Amish Family Diabetes Study (AFDS) (Fu *et al.* 2004), and Pharmacogenomics of Antiplatelet Intervention (PAPI) (Lewis *et al.* 2013). Details of study design, recruitment, and phenotyping have been previously described (Fu *et al.* 2004; Lewis *et al.* 2013; Lu *et al.* 2015). SNPs (rs3798220 and rs10455872) were genotyped in 2919 Amish subjects using TaqMan Allelic Discrimination Assay (Applied Biosystems) and 146 subjects were recruited into the present study (Figure 1). Since carriers of minor alleles for both rs3798220 and rs10455872 are infrequent (MAF: 0.009 and 0.022, respectively), we recruited 31 carriers of rs3798220 and 42 carriers of rs10455872. Noncarrier control subjects were matched, as closely as possible, to the carriers according to gender and age (±3 yr). To exclude double mutation of rs3798220 and rs10455872 in recruited subjects, all of the carriers and noncarriers of rs3798220 had the same genotype (*AA*) for rs10455872. Likewise, all of the rs10455872 individuals had the same genotype (*TT*) for rs3798220. The study was approved by the institutional review board of the University of Maryland, Baltimore, and all participants provided written informed consent. The methods were carried out in accordance with the approved guidelines.

### Plasma samples

Venous blood was drawn after an overnight fast. Four milliliters of blood was collected from each individual in vacutainer tubes containing EDTA [for Lp(a) and plasminogen measurement] or 3.2% sodium citrate (for ECLA), respectively. Plasma samples were subsequently separated by centrifugation at 2000 × *g* for 15 min at 4°. The plasma supernatant for ECLA measurement was recentrifuged at 2000 × *g* for 15 min at 4° to remove any residual platelets. Multiple aliquots of plasma were stored at −80° until assays were performed.

### ELISA

Quantitative determination of Lp(a) and plasminogen antigen levels in human plasma samples was performed by an enzyme-linked immunosorbent assay (ELISA) according to the manufacturer’s instruction (Assaypro, St. Charles, MO). Fifty microliters of standard or plasma using EDTA as an anticoagulant, diluted with dilution buffer (1:8000 dilution for Lp(a); 1:20,000 dilution for plasminogen), were added into each well of 96-well plates precoated with a polyclonal antibody specific for human Lp(a)/plasminogen. Wells were then incubated with the biotinylated polyclonal antibody specific for human Lp(a)/plasminogen, and was recognized by a streptavidin-peroxidase conjugate, and then a peroxidase enzyme substrate. After the reaction was stopped, the absorbance at 450 nm was read on a VICTOR X3 Multilabel Plate Reader (PerkinElmer, Waltham, MA). The standard curves were generated by polynomial regression analysis. Lp(a) and plasminogen levels were expressed in micrograms per milliliter.

### Euglobulin clot lysis assay

ECLA was modified from the assay described by Manco-Johnson (Smith *et al.* 2003). Briefly, the euglobulin fraction was prepared by adding 350 µl of plasma into 6.3 ml of 0.016% acetic acid solution, and then samples were incubated in an ice bath for 10 min. The precipitated euglobulin fraction was then resuspended with 350 µl of borate buffer (154 mM sodium chloride, 2.6 mM sodium borate, pH 9.0). One hundred microliters of sample was pipetted in triplicate (two wells for measurement and one well for blank) into wells of prewarmed 37° untreated 96-well plates, followed by addition of 100 µl of 0.025 mM CaCl_2_ to each well (100 µl of ddH_2_O added to the blank wells). The plate was read on a VICTOR X3 Multilabel Plate Reader (PerkinElmer) at 405 nm at 3-min intervals for 10 hr and the temperature was maintained at 37°.

### Liver samples

Seventy-six wedge biopsy liver samples were obtained from Caucasian patients undergoing open or laparoscopic Roux-en-Y gastric bypass operations or laparoscopic adjustable gastric banding procedures for extreme obesity or its comorbid medical problems at Geisinger Medical Center, Danville, PA (Lu *et al.* 2015; Still *et al.* 2011). Simply, we genotyped 1328 Caucasian patients for rs3798220 and rs10455872 using TaqMan Allelic Discrimination Assay (Applied Biosystems). These liver samples were selected according to the carrier and noncarrier status for rs3798220 (*N* = 19: 19 for genotypes *TT* and *CT*) and rs10455872 (*N* = 20: 20: 3 for *AA: AG: GG*), and also matched for gender, age, and BMI (Figure 1) (Lu *et al.* 2015).

### qRT-PCR

Total liver RNA was extracted by TRIzol (Invitrogen, Grand Island, NY) according to the manufacturer’s instructions. The resulting RNA was subjected to DNAse digestion using RNase-free DNase Set (Qiagen, Valencia, CA) and purification using RNeasy MinElute Cleanup Kit (Qiagen). The RNA quantity and quality were determined using a ND1000 nanodrop spectrophotometer, and 1 µg of total RNA was reverse-transcribed using a Transcriptor First Strand cDNA Synthesis Kit (Roche, Indianapolis, IN). Real-time PCR was performed using TaqMan gene expression assay primers and probes [assay ID: Hs00264877_m1 for human *PLG*; Hs99999903_m1 for human β-actin (*ACTB*)]. Steady-state mRNA levels were determined by two-step quantitative real-time PCR (qRT-PCR) using the LightCycler 480 (Roche) and TaqMan probe/primer sets (Applied Biosystems, Grand Island, NY). Relative expression of mRNAs was determined after normalization with the reference gene *ACTB* levels using LightCycler 480 software 1.5.

### Statistical analysis

Data were presented as mean ± SE for the clinical profile, qRT-PCR, ELISA, and ECLA. A two-tailed Student’s *t*-test was used to evaluate the statistical significance for all assays except for the *PLG* mRNA expression of rs10455872 in which ANOVA was applied (GraphPad Software, La Jolla, CA). A *P* value <0.05 was considered statistically significant. Pairwise linkage disequilibrium (LD) statistics (D′ and *r*^2^) were calculated with Haploview version 4.2 (https://www.broadinstitute.org/scientific-community/science/programs/medical-and-population-genetics/haploview/haploview) (Barrett *et al.* 2005).

All statistical tests were two-sided. Power estimates in 42 (rs10455872) and 31 (rs3798220) pairs of subjects were calculated using the Power and Sample Size Calculation software (version 3.1.2) (Dupont and Plummer 1990). Prior data indicate that the difference in the response of matched pairs is normally distributed with standard deviation. We will be able to detect a true difference in the mean response of matched pairs of −0.443 or 0.443 and −0.520 or 0.520, respectively, with probability (power) 0.8. The type I error probability associated with this test of the null hypothesis that this response difference is zero is 0.05.

### Data availability

Both the genotypic and phenotypic information for rs3798220 and rs10455872 used in this study are listed in Supplemental Material, Table S1 and Table S2, respectively.

## Results

### Clinical characteristics of the OOA subjects

To evaluate whether *LPA* genetic variants affect plasma plasminogen levels and fibrinolysis, we successfully genotyped rs3798220 and rs10455872 in 2919 Amish subjects and identified 76 carriers of rs3798220 and 125 carriers of rs10455872. We recruited 146 age- and sex-matched subjects according to their genotype status. The mean age was 46.74 yr old, and 46.76% of subjects were female. Since carriers of minor alleles for both rs3798220 and rs10455872 were infrequent (MAF: 0.009 and 0.022, respectively) in OOA, no minor allele homozygotes were recruited. The clinical characteristics of the 146 OOA subjects are summarized in Table 1. Briefly, study subjects were relatively healthy adults, and the mean levels of age, BMI, blood pressure, HDL, LDL, TG, hematocrit, red blood cell count, white blood cell count, and platelet count were not significantly different between the carriers and noncarriers of the two *LPA* genetic variants rs3798220 and rs10455872.

**Table 1:** Clinical profile for the OOA subjects by genotype

	rs3798220			rs10455872		
	*TT* (*n* = 31)	*CT* (*n* = 31)	*P* Value	*AA* (*n* = 42)	*AG* (*n* = 42)	*P* Value
Gender	Male: 14	Male: 14		Male: 24	Male: 24	
	Female: 17	Female: 17		Female: 18	Female: 18	
Age (years)	47.87 ± 2.34	46.32 ± 2.42	0.659	46.62 ± 2.13	46.38 ± 2.13	0.937
BMI (kg/m^2^)	29.24 ± 1.04	28.41 ± 1.11	0.594	28.38 ± 0.92	26.70 ± 0.63	0.138
SBP (mmHg)	117.07 ± 2.29	119.55 ± 2.93	0.511	120.19 ± 1.62	118.71 ± 1.63	0.523
DBP (mmHg)	75.07 ± 1.59	72.74 ± 1.83	0.346	76 ± 1.17	74.31 ± 1.30	0.337
HDL (mg/dl)	48.53 ± 2.32	53.97 ± 2.29	0.103	52.95 ± 2.52	58.17 ± 2.42	0.142
LDL (mg/dl)	127.77 ± 6.86	145.35 ± 7.32	0.087	130.6 ± 6.69	137.82 ± 6.83	0.454
TG (mg/dl)	86.17 ± 8.86	83.13 ± 9.72	0.820	77.93 ± 6.76	78.93 ± 10.09	0.935
Hct (%)	40.07 ± 0.63	41.03 ± 0.55	0.279	41.24 ± 0.50	40.64 ± 0.50	0.411
RBC count (*n* × 100,000)	4.52 ± 0.07	4.64 ± 0.07	0.278	4.56 ± 0.05	4.48 ± 0.06	0.377
WBC count (*n* ×1000)	5.42 ± 0.25	6.18 ± 0.31	0.066	5.29 ± 0.18	5.62 ± 0.22	0.252
Platelet count (*n* × 1000)	240.8 ± 9.88	236.77 ± 8.63	0.773	235.8 ± 7.82	253.56 ± 8.43	0.135

Data are presented as mean ± SE.

### Association between LPA genetic variants and plasma Lp(a) levels

**Figure 2:**
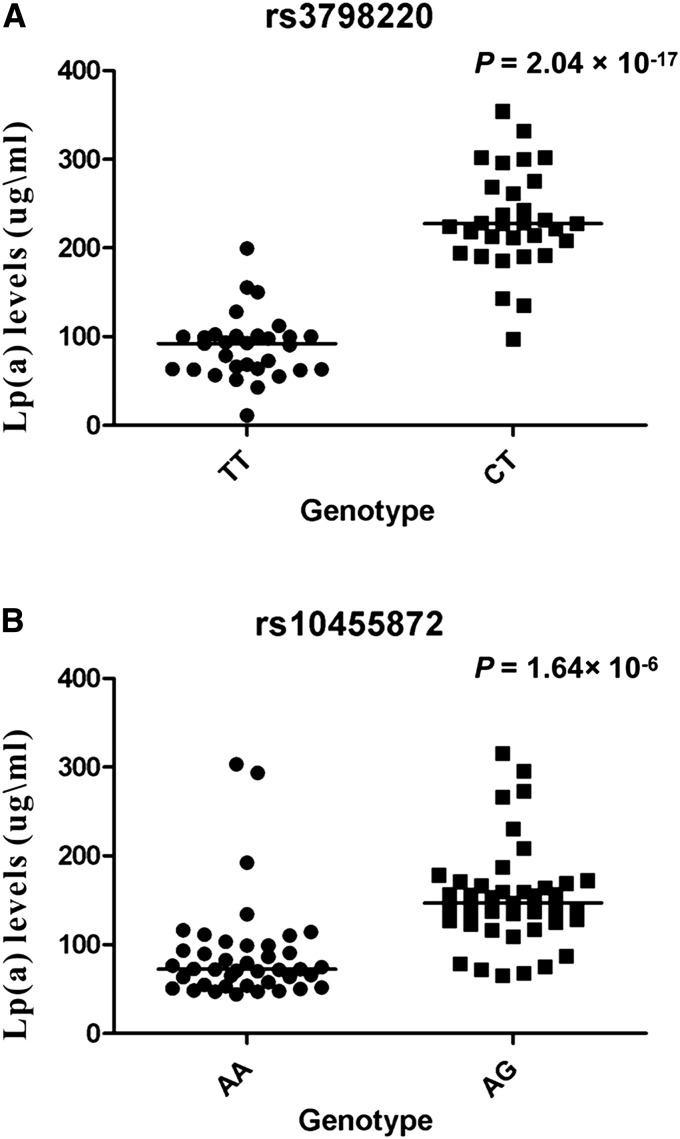
Plasma Lp(a) levels among the OOA subjects for the rs3798220 genotype (A) and the rs10455872 genotype (B). A scattergram is shown for individuals of each genotype with the median represented by a black line respectively.

To confirm whether *LPA* genetic variants rs3798220 and rs10455872 significantly influence plasma Lp(a) levels, we measured plasma Lp(a) levels by an ELISA (Assaypro) in our 146 Amish participants. We observed that plasma Lp(a) levels were significantly higher for the carriers of both rs3798220 and rs10455872 compared to the noncarriers. Specifically, the Lp(a) levels in carriers of rs3798220 were 2.62 times higher than the Lp(a) levels in noncarriers (carriers: 230.96 ± 10.06 µg/ml *vs.* noncarriers: 88.23 ± 6.55 µg/ml, *P* = 2.04 × 10^−17^) (Figure 2A). Similarly, the Lp(a) levels in carriers of rs10455872 were 1.73 times higher than the Lp(a) levels in noncarriers (carriers: 154.01 ± 9.00 µg/ml *vs.* noncarriers: 88.98 ± 8.76 µg/ml ; *P* = 1.64× 10^−6^) (Figure 2B).

### Relationship between LPA genetic variants and plasma plasminogen levels

**Figure 3:**
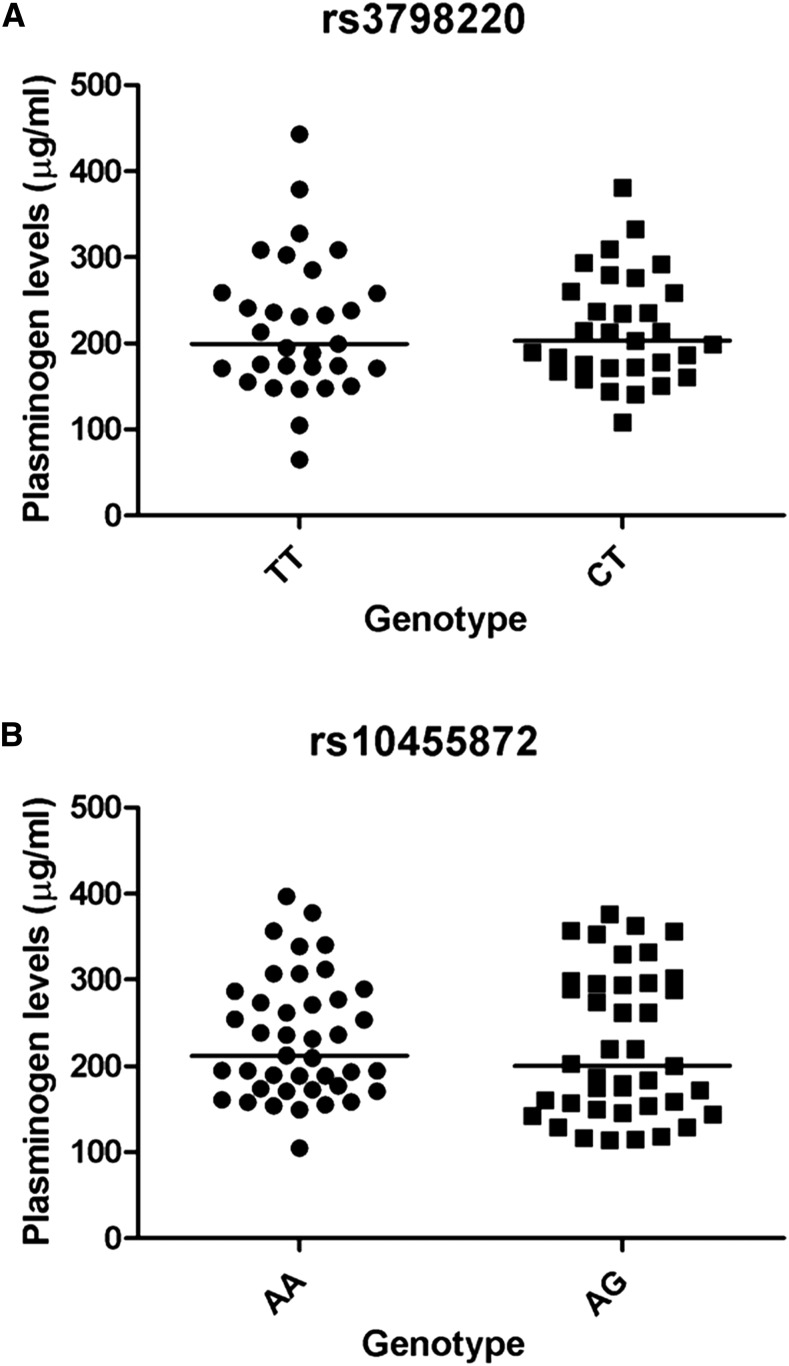
Plasma plasminogen levels among the OOA subjects for the rs3798220 genotype (A) and the rs10455872 genotype (B). A scattergram is shown for individuals of each genotype with the median represented by a black line respectively.

To investigate whether *LPA* genetic variants rs3798220 and rs10455872 significantly affect plasma plasminogen levels, we measured plasma plasminogen levels by ELISA (Assaypro) in the Amish cohort. We observed that the plasminogen levels in the carriers of rs3798220 and rs10455872 were very close to the levels in the noncarriers (carriers: 217.02 ± 11.34 µg/ml *vs.* noncarriers: 219.47 ± 14.49 µg/ml, and carriers: 223.96 ± 13.02 µg/ml *vs.* noncarriers: 232.09 ± 10.99 µg/ml, respectively). The plasminogen levels in the carriers were not significantly different from the levels observed in the noncarriers of rs3798220 (*P* = 0.89) (Figure 3A) and rs10455872 (*P* = 0.63) (Figure 3B).

### Relationship between LPA genetic variants and fibrinolysis

**Figure 4:**
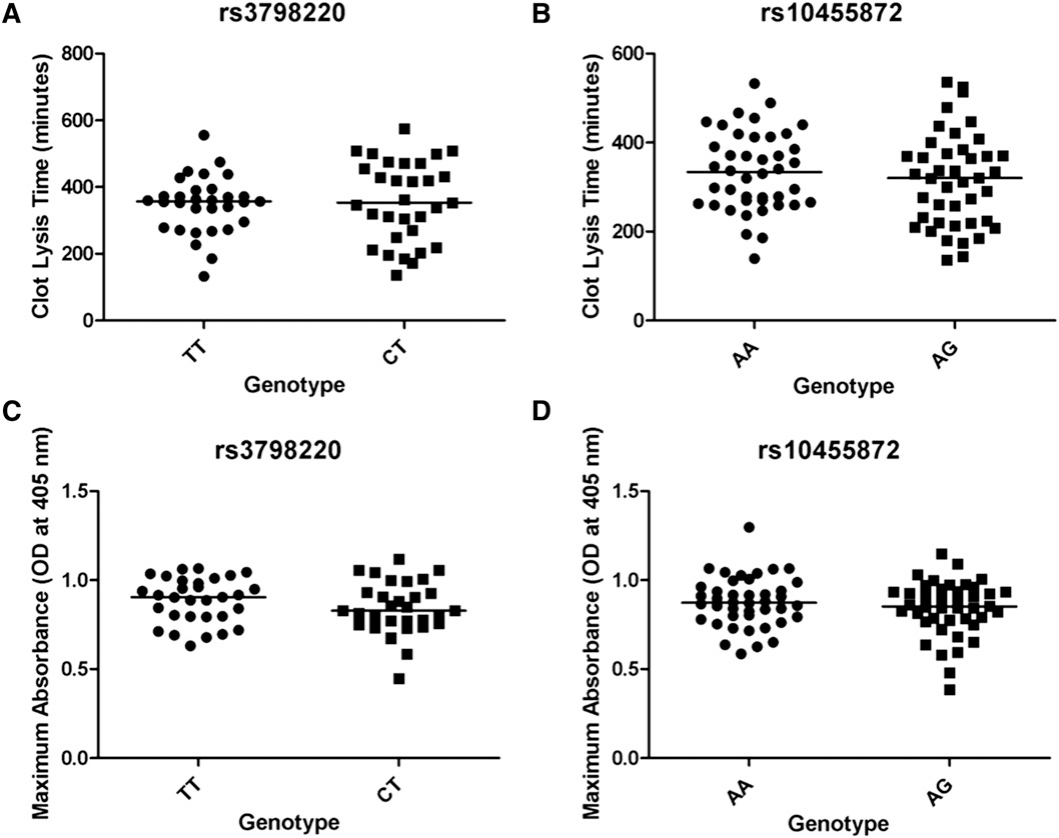
Results of ECLA for the OOA subjects with different genotypes. Clot lysis times measured in minutes for the rs3798220 genotype (A) and for the rs10455872 genotype (B). Maximum absorbance (OD at 405 nm) measured for the rs3798220 genotype (C) and the rs10455872 genotype (D). A scattergram is shown for individuals of each genotype with the median represented by a black line respectively.

In addition to assessing genotype-specific differences in Lp(a) and plasminogen levels, we investigated whether *LPA* SNPs rs3798220 and rs10455872 were associated with fibrin clot lysis. Fibrinolysis was assessed using a euglobulin clot lysis time (ECLT) and maximum absorbance (OD at 405 nm) by the ECLA. We observed no difference in ECLT between carriers and noncarriers of rs3798220 (*CT* *vs.* *TT*, 357.19 ± 21.68 min *vs.* 347.26 ± 15.36 min; *P* = 0.71, Figure 4A) or rs10455872 (*AG* *vs.* *AA*, 315.91 ± 16.16 min *vs.* 334.08 ± 13.67 min; *P* = 0.39, Figure 4B), respectively. In addition, the maximum absorbance in carriers of rs3798220 and rs10455872 were not significantly different from the maximum absorbance in the noncarriers [rs3798220 *CT* *vs.* *TT*, 0.84 ± 0.027 *vs.* 0.89 ± 0.023; *P* = 0.18 (Figure 4C) and rs10455872 *AG* *vs.* *AA*, 0.84 ± 0.024 *vs.* 0.88 ± 0.022; *P* = 0.26 (Figure 4D)].

### PLG mRNA expression in the liver

**Figure 5:**
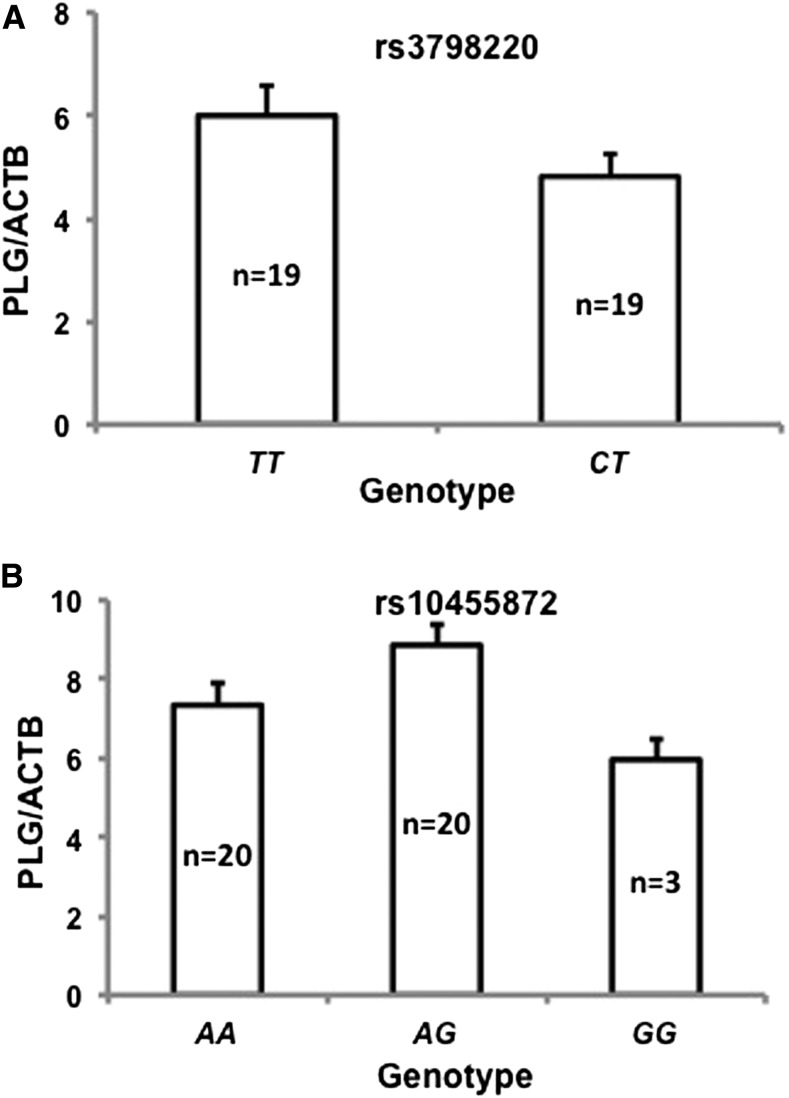
Plasminogen mRNA expression in the liver. (A) *PLG* mRNA expression in the subjects with the rs3798220 genotype; (B) *PLG* mRNA expression in the subjects with the rs10455872 genotype. Data are presented as mean ± SE.

Given the close proximately of rs3798220 and rs10455872 with the *PLG* gene, we tested whether these variants had any influence on *PLG* mRNA expression in the liver. Total RNA was extracted from liver samples derived from Caucasian patients undergoing bariatric weight loss procedures for extreme obesity or related comorbid medical conditions (Lu *et al.* 2015). Patients were selected according to the genotype for rs3798220 (*N* = 19 and 19 for genotypes *TT* and *CT*, respectively) and rs10455872 (*N* = 20, 20, and 3 for genotypes *AA*, *AG*, and *GG*) and matched for age and gender. We measured the *PLG* mRNA expression by qRT-PCR and found that there was no statistically significant difference in expression between carriers of rs3798220 and noncarriers (*TT*, 6.00 ± 0.59; *CT*, 4.82 ± 0.44; *P* = 0.12) (Figure 5A). Similarly, no difference in plasminogen RNA expression was observed between rs10455872 genotype groups (*AA*: 7.35 ± 0.58, *AG*: 8.87 ± 0.55, *GG*: 5.97 ± 0.52, *P =* 0.06; *AG* + *GG* *vs.* *AA*, *P* = 0.15) (Figure 5B).

### Linkage disequilibrium analysis

**Figure 6:**
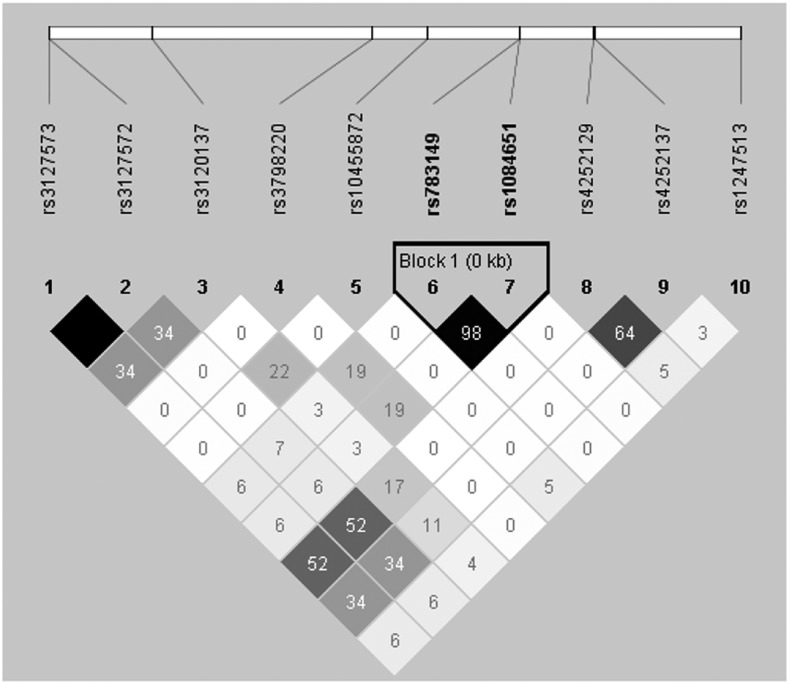
Linkage disequilibrium pattern of rs3798220, rs10455872, and top *PLG* association SNPs in chromosome 6q25–26.

We performed LD analysis for rs3798220, rs10455872, and the top SNPs associated with plasminogen on chr.6q26 in previous GWAS (Ma *et al.* 2014). The result of LD analysis showed these two SNPs did not have high LD with any of the top SNPs for plasminogen (*r*^2^ from 0 to 0.227, Figure 6).

## Discussion

Lp(a) has a causal role in the development of multiple cardiovascular disorders (Arsenault *et al.* 2014; Clarke *et al.* 2009; Kamstrup *et al.* 2009) and is recognized as having both atherogenic and thrombogenic characteristics (Caplice *et al.* 2001; Enas *et al.* 2006; Gavish *et al.* 1989; Marcovina and Koschinsky 2003). *LPA* SNPs rs3798220 and rs10455872 are consistently associated with Lp(a) levels and result in increased risk for cardiovascular diseases (Arsenault *et al.* 2014; Clarke *et al.* 2009; Kutikhin *et al.* 2014; Thanassoulis *et al.* 2013). In the present study, we investigated the effects of these variants not only on their impact on Lp(a) levels but also on their potential effect on thrombogenicity in order to provide novel insights regarding the mechanism(s) by which *LPA* variants confer CVD susceptibility. To our knowledge this study represents the first investigation to simultaneously assess the impact of these variants on both Lp(a) levels and markers of thrombosis (*i.e.*, plasminogen levels and fibrinolysis). Consistent with previous results, we observed that both of these SNPs significantly impact Lp(a) levels; however, we observed no evidence to suggest that these variants influence plasminogen levels. In addition, we extend these finding to show that neither rs3798220 nor rs10455872 impact fibrinolytic activity or *PLG* mRNA expression in the liver.

The OOA community is a genetically well-defined Caucasian founder population. More than 95% of the current Lancaster Amish population can trace their ancestry to one of seven founder couples (Agarwala *et al.* 1999, 2001). The Amish today are a rural, mostly farming, community, and strong religious beliefs help them to maintain the sect as a distinct and closed entity (Cross 1976). They are relatively homogenous in terms of genetic ancestry, environment, and lifestyle characteristics, which minimizes the risk of potentially confounding variables and makes the OOA a particularly advantageous group for genetic studies. In this investigation, we have genotyped the two genetic variants of rs3798220 and rs10455872 in 2919 Amish subjects. To maximally limit confounding factors, we recruited subjects according to carrier and noncarrier status for rs3798220 and rs10455872, and matched for gender and age. The minor allele frequencies of rs3798220 and rs10455872 in the OOA are 0.9% and 2.2%, respectively (Lu *et al.* 2015), which is relatively lower than the minor allele frequencies (2 and 7%, respectively) in outbred European populations (Clarke *et al.* 2009). We did not recruit subjects who were homozygous for the minor allele of these variants in OOA. Fortunately, these two genetic variants are dominant genetic disorders meaning that a single allele can control whether the disease develops. We can recruit heterozygotes or/and minor allele homozygotes to study biological function. Consistent with previous studies, the plasma Lp(a) levels were significantly higher for carriers of rs3798220 or rs10455872 compared to noncarriers in the OOA subjects (Clarke *et al.* 2009; Laschkolnig *et al.* 2014; Lu *et al.* 2015).

Plasminogen is the proenzyme precursor of the primary fibrinolytic protease plasmin, which has an important role in tissue remodeling and blood clot removal after injury (Chapin and Hajjar 2015). Genetic variants in the *LPA* gene were identified as significant contributors to plasminogen levels by GWAS (Ma *et al.* 2014). Our data showed that rs3798220 and rs10455872 are not significantly associated with the plasma plasminogen levels. This finding is consistent with a previous report that rs10455872 was the most significantly associated SNP with Lp(a) levels but was not significantly associated with plasminogen levels in healthy subjects (Ma *et al.* 2014). Furthermore, our present study clarified for the first time that another Lp(a)-associated SNP, rs3798220, also was not significantly associated with plasminogen levels. Linkage disequilibrium analysis for rs3798220 and rs10455872 and the top SNPs associated with plasminogen on Chr. 6q26 in previous GWAS showed that the investigated SNPs did not have high LD with any PLG-associated SNPs. Our data suggest that the Lp(a)-associated variants rs3798220 and rs10455872 in the *LPA* gene are not the major genetic determinants of plasma plasminogen levels.

Since apo(a) can bind to fibrin but has no proteolytic activity, it has been hypothesized that apo(a) competes with plasminogen in circulation and may attenuate fibrinolytic function (Angles-Cano *et al.* 2001; Loscalzo *et al.* 1990). We investigated whether genetic variation in *LPA* (*i.e.*, rs3798220 and rs10455872) influences fibrinolysis and found no difference in clot lysis time and maximum absorbance between carriers and noncarriers of both rs3798220 and rs10455872, indicating that clot formation and clot lysis are not affected by these two SNPs. Of note, however, Undas *et al.* reported decreased clot permeability and longer clot lysis time in both healthy subjects and patients with myocardial infarction that have increased Lp(a) levels [a cutoff value of Lp(a) 300 µg/ml] as a result of small apo(a) size isoforms (Undas *et al.* 2006). Recently, Rowland *et al.* found that the *LPA* genetic variant rs3798220 was associated with decreased clot permeability and longer clot lysis time among Caucasians, but was associated with increased clot permeability and shorter clot lysis among non-Caucasians (Rowland *et al.* 2014). Mansson *et al.* however, reported that Lp(a) plasma levels had no effect on clot lysis time in diabetic subjects and normal controls (Mansson *et al.* 2014). These discordant results may be caused by: (1) differing study designs; (2) different measure methods; (3) small sample size; and/or (4) differences in population characteristics. In the present study, we chose relatively homogenous healthy OOA subjects that were matched with regards to sex, age, and the other lipid traits. We had a relatively large sample size and measured fibrinolysis using an ECLA method without addition of human thrombin and recombinant tissue plasminogen activator (rtPA). We identified that rs3798220 and rs10455872 were not major genetic determinants for fibrinolysis.

The *LPA* and *PLG* genes are adjacently located on chromosome 6 and have a high degree of structural homology, with the *LPA* gene believed to be generated from the duplication of the *PLG* gene (McLean *et al.* 1987). Since the liver is the major site of both *LPA* and *PLG* mRNA synthesis, it is possible that there is coregulation between hepatic *LPA* and *PLG* mRNA expression. We determined *PLG* mRNA levels using total RNA extracted from liver samples as described in our previous study (Lu *et al.* 2015). Previously, we determined that levels of *LPA* mRNA were higher in carriers of rs10455872 as compared to noncarriers, and were not different between the carriers and noncarriers of rs3798220 (Lu *et al.* 2015). In the present study, no significant differences in *PLG* mRNA levels were observed between the carriers and noncarriers of rs10455872 and rs3798220. In addition, a previous animal study reported that there was no correlation between hepatic *LPA* and *PLG* mRNA levels, suggesting an independent regulation for *LPA* and *PLG* mRNA expression (Ramharack *et al.* 1996).

There are some limitations of this study that we acknowledge. According to power estimation, we have nearly 100% power to detect a large effect size and 80% power to detect a medium effect size, but we do not have power to detect small effect of rs3798220 and rs10455872 on variation of plasma plasminogen, plasminogen mRNA expression, and change of fibrinolysis, which may lead to the negative findings. However, our data show these two variants are not the major genetic determinants of plasminogen levels and fibrinolysis although it is known that they contribute to the risk of cardiovascular diseases.

In conclusion, our data indicate that two genetic variants in the *LPA* gene, rs3798220 and rs10455872, are significantly associated with elevated Lp(a) concentration, but not significantly associated with variation in plasma plasminogen concentration. The increased levels of plasma Lp(a) with these genetic variants do not affect fibrinolysis in healthy subjects. Moreover, these two Lp(a)-associated genetic variants were not associated with *PLG* mRNA expression in the liver. These two Lp(a)-associated variants of rs3798220 and rs10455872 in the *LPA* gene are not the major genetic determinants of fibrinolysis, *PLG* mRNA expression, and plasminogen levels.

## Supplementary Material

Supplemental Material
