# Supplementary material for: Effect of Two Lipoprotein (a)-Associated Genetic Variants on Plasminogen Levels and Fibrinolysis
Source: G3 (Bethesda). 2016 Sep 6;6(11):3525–32. doi: 10.1534/g3.116.034702 (PMC5100851; doi:10.1534/g3.116.034702)
Supplement: Supplemental Material [file supp_6_11_3525__index.html]

Effect of Two Lipoprotein (a)-Associated Genetic Variants on Plasminogen Levels and Fibrinolysis — Supplemental Material 

# Effect of Two Lipoprotein (a)-Associated Genetic Variants on Plasminogen Levels and Fibrinolysis

## Supplemental Material for Wang *et al.*, 2016

**Files in this Data Supplement:**

- Table S1 - The genotypic and phenotipic information for rs3798220. (.xlsx, 17 KB)
- Table S2 - The genotypic and phenotipic information for rs10455872. (.xlsx, 22 KB)
